# Exploring the Fungal Community and Its Correlation with the Physicochemical Properties of Chinese Traditional Fermented Fish (Suanyu)

**DOI:** 10.3390/foods11121721

**Published:** 2022-06-13

**Authors:** Haixin Sun, Xiaochang Liu, Liwen Wang, Yaxin Sang, Jilu Sun

**Affiliations:** 1 College of Food Science and Technology, Hebei Agricultural University, Baoding 071001, China; haixinsun1405@163.com (H.S.); liwen.wang@lincolnuni.ac.nz (L.W.); sangyaxin@sina.com (Y.S.); 2 Institute of Animal Sciences, Chinese Academy of Agricultural Sciences, Beijing 100093, China; lxc_cau@163.com

**Keywords:** Suanyu, physicochemical properties, fungal community, high-throughput sequencing, biogenic amine

## Abstract

Suanyu is a traditional natural fermented fish product from Southwest China that contains very complex microflora. The main purpose of this study was to explore the fungal community and its relationship with the physicochemical properties of Suanyu. The fungal community structure of Suanyu from the main provinces (Guizhou and Hunan) was studied via high-throughput sequencing. The correlation between dominant fungi and physicochemical characteristics was analyzed via Spearman’s correlation coefficient. The results showed that the pH value, total volatile base nitrogen content, and thiobarbituric acid reactive substance content ranges of Suanyu samples were 4.30–5.50, 17.11–94.70 mg/100 g, and 0.61 to 3.62 mg/kg, respectively. The average contents of total volatile base nitrogen, thiobarbituric acid reactive substance, and total BAs in Suanyu from Guizhou were lower than those from Hunan. The main BAs were phenethylamine, putrescine, cadaverine, histamine, and tyramine. Ascomycota was the dominant fungal phylum, and *Kodamaea*, *Debaryomyces*, *Wallemia*, *Zygosaccharomyces,* and unclassified *Dipodascaceae* were the dominant fungal genera in different samples. Moreover, high abundance levels of *Kodamaea* and *Zygosaccharomyces* were found in Suanyu from Guizhou. According to the correlation analysis, *Kodamaea* and *Zygosaccharomyces* were negatively correlated with TBARS (R^2^ = −0.43, −0.51) and TVBN (R^2^ = −0.37, −0.29), and unclassified *Dipodascaceae* was significant negatively correlated with tyramine (R^2^ = −0.56). This study expands the understanding of the fungal community and the fermentation characteristics of the dominant fungi in Suanyu.

## 1. Introduction

Suanyu is a traditional fermented fish product from Southwest China. Due to its characteristic flavor and high nutritional value, it is popular with local consumers, specifically in Hunan and Guizhou provinces [1]. Traditional Suanyu is usually prepared in small family workshops without adding a standardized starter culture. The manufacturing process consists of mixing whole freshwater fish (carp, white amur, or crucian carp) with cooked rice, spices, and salt, with fermentation in anaerobic conditions for at least 1–2 months [2,3].

The spontaneous fermentation of Suanyu is a complex microbial succession process. Microorganisms have distinct effects on fermentation, such as accelerating the protein degradation, shortening the fermentation time, promoting the formation of precursor flavor substances such as organic acids and free amino acids, and improving the safety of Suanyu [4]. In order to improve the quality of Suanyu, it is necessary to explore the microorganisms in Suanyu and their effect on the quality [4,5,6]. Previous studies have extensively explored the bacterial community in Suanyu and isolated some autochthonous strains that can be used as starter cultures, including *Lactobacillus plantarum* and *Staphylococcus xylosus* [1,5,7,8]. Starter cultures isolated from Suanyu play a positive role in lipolysis, lipid oxidation, and flavor improvement [8,9,10]. However, some isolated microorganisms have the potential to produce biogenic amines. Biogenic amines are low-molecular weight nitrogenous organic compounds, which are widely found in fermented foods and are mainly generated via the decarboxylation of amino acids by microorganisms [11,12]. An appropriate amount of biogenic amines has a regulating effect on physiological activities [11], while an excess of biogenic amines (approximately 1000 ppm) can cause headache, heart palpitations, diarrhea, hypertension, and other symptoms [13,14]. Currently, there are no established standards or regulations for Suanyu to limit the biogenic amines levels. Meng et al. [15] revealed the important role of *Enterobacteriaceae* in producing amines in Suanyu. Zeng et al. [16] inoculated mixed autochthonous starter cultures in low-salt fermented Suanyu and found that the contaminant microorganisms were inhibited and the content of biogenic amines decreased.

These previous studies mainly focused on the bacterial community in Suanyu and paid little attention to the fungal community. However, *Debaryomyces*, *Candida*, *Saccharomyces*, and *Torulospora* are common yeasts in several traditional fermented fish products, and yeasts have an important influence on the taste, aroma, texture, and nutritional value of fermented products [17]. Therefore, this study aimed to analyze the fungal communities in traditional Suanyu produced in two main provinces (Guizhou and Hunan) via high-throughput sequencing methods, along with the correlations between dominant fungi and physicochemical properties of Suanyu with Spearman’s correlation coefficient. This study will be helpful in understanding the role of the fungal community in the traditional fermentation of Suanyu and in instructing the isolation of potential strains as starters of Suanyu.

## 2. Materials and Methods

### 2.1. Collection and Pretreatment of Suanyu Sample

**Figure 1:**
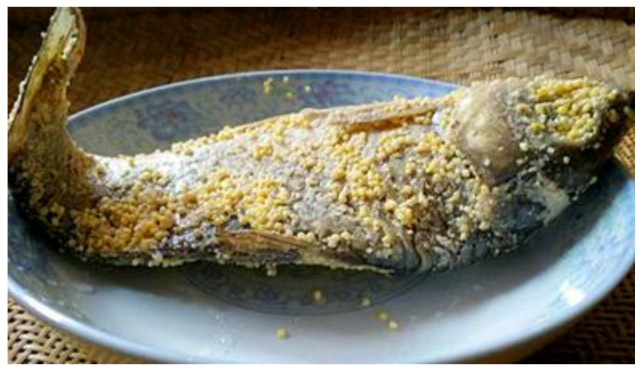
Suanyu samples after fermentation.

Suanyu samples were collected from local farmer’s homes in Guizhou and Hunan provinces in China (Table 1). The fish used in these samples were fresh carp (0.5–1.5 kg), harvested in 2020, which were fermented using a solid-state process. The carp were mixed with ingredients after removing the gills and guts and were cured at 4 °C for 1–2 days. The ingredients included salt, sucrose, cinnamon, star anise, chili powder, and pepper. The proportion was controlled by the local farmers. The samples were dried in the sun. Thereafter, the fish were mixed with rice, roasted corn, or millet. Finally, the above fish were placed in ceramic pots with a water seal and fermented at natural temperatures (22–25 °C) for 1–2 months (Figure 1). Eight Suanyu samples were collected from Guizhou, numbered as G1 to G8. Other samples from Hunan were marked as H1 to H8. Samples were kept in sterile bags, stored in ice boxes, and transported to the laboratory at 0–4 °C.

A total of 16 Suanyu samples were analyzed. The entrails, spines, and scales were removed and the carp meat was mixed using a blender and frozen at −80 °C (Haier, DW-86L338J, Qingdao, China) for further analysis.

### 2.2. Physicochemical Analysis of Suanyu

The pH value was analyzed according to the method used by Zang et al. [18] with slight modifications. The meat from Suanyu samples (1 g) and 9 mL distilled water were homogenized under 10,000 r/min for 1 min. The pH value of the mixture was determined using a pH meter (PHS-3DW, Bridgesi, Hefei, China).

The content of total volatile base nitrogen (TVBN) was determined according to the method used by Kjeldahl [19] with slight modifications and expressed as mg/100 g. In brief, the meat from the Suanyu samples (10 g) and 100 mL distilled water were mixed and centrifuged at 2000× *g* and 4 °C for 5 min, then the supernatant was distilled with 0.1% (*w*/*v*) MgO for 10 min. The absorption liquid (including 2% H_3_BO_3_ (*w*/*v*) and a mixed indicator (1 g/L methyl red in absolute ethanol to 1 g/L bromocresol green in absolute ethanol at a 1:5 ratio)) was titrated with 0.1 M HCl for quantification.

The thiobarbituric acid reactive substance (TBARS) value was measured as described by Gao et al. [9] with slight modifications. Briefly, the meat from Suanyu samples (5 g) was mixed with 50 mL of trichloroacetic acid (10%) and incubated at 50 °C for 30 min, then the supernatant was filtered through filter paper. The final mixture (the supernatant and thiobarbituric acid (20 mM) were mixed at a 1:1 ratio)) was placed in a boiling water bath for 20 min. The absorbance was measured at 532 nm. The results were calculated from an MDA (malondialdehyde) standard curve and expressed as mg/kg.

### 2.3. Biogenic Amines Determination

The compositions of biogenic amines in Suanyu were measured using the method used by Li et al. [20] and Li et al. [21]. Suanyu samples (2.5 g) were homogenized with 25 mL of 0.1 M HCl in a high-speed blender (FSH-2; Guohua Co., Changzhou, China) at 16,000 r/min for 1 min and the mixture was centrifuged at 4298× *g* and 4 °C for 30 min. Then, 200 μL NaOH (2 M), 300 μL of saturated solution of NaHCO_3_, and 1 mL dansyl chloride (10 mg/mL) were added to the 1 mL of supernatant and incubated at 40 °C for 45 min under dark conditions. After adding 100 μL of ammonium hydroxide, the mixture was left for 30 min under dark conditions, then adjusted to 5 mL with acetonitrile and filtered through a 0.22 μm organic phase needle filter. The injection volume was 20 μL for analysis. All tests were performed in triplicate. Bas were analyzed using the Waters high-performance liquid chromatography (HPLC) system (Watts Technology Shanghai, Co., Shanghai, China) with a WondaSil C18 chromatographic column (4.6 mm × 250 mm). Solvent A (0.1 M ammonium acetate) and solvent B (acetonitrile) were selected as the mobile phase. The flow rate was 1.0 mL/min and the temperature of column was 40 °C. The gradient elution program was as described by Li et al. [21].

### 2.4. DNA Extraction and PCR Amplification

Total genomic DNA was extracted from 16 samples with the E. Z.N.A.^®^ soil DNA kit (Omega Bio-tek, Norcross, GA, USA) following the manufacturer’s instructions. Extracted DNA was assessed on a 2% (*w*/*v*) agarose gel. The ITS1-ITS2 gene of the fungi was amplified with the forward primer ITS1F (5′- CTTGGTCATTTAGAGGAAGTAA-3′) and the reverse primer ITS2R (5′- GCTGCGTTCTTCATCGATGC-3′) using an ABI GeneAmp^®^ 9700 PCR thermal cycler (ABI, Foster, CA, USA) [18]. The reaction system contained 10 × buffer 2 μL, 2.5 mM dNTPs 2 μL, forward primer (5 μM) 0.8 μL, reverse primer (5 μM) 0.8 μL, rTaq polymerase 0.2 μL, BSA 0.2 μL, template DNA 10 ng, and ddH2O to a final volume of 20 μL. The reaction conditions were as follows: initial denaturation at 95 °C for 3 min, followed by 35 cycles (95 °C for 30 s, 55 °C for 30 s, 72 °C for 45 s) and a final extension at 72 °C for 10 min.

### 2.5. Illumina MiSeq Sequencing

The PCR products were sequenced at Shanghai Meiji Biotechnology Co., Ltd., Shanghai, China, after purification and recovery via 2% agarose gel electrophoresis.

### 2.6. Processing of Sequencing Data

The PE 2 × 300 bp library was constructed and the high-throughput sequencing of fungal sequences was qualified on an Illumina MiSeq platform. Based on the overlap relationship, paired-end reads were filtered using Trimmomatic and generated using Flash (version 1.2.11). The effective sequences with ≥97% similarities were clustered using UPARSE (version 7.0.1090). The alpha diversity was evaluated using Mothur (version 1.30.2).

### 2.7. Statistical Analysis

Experiments were performed in triplicate. The statistical software programs employed were SPSS 23 and Microsoft Excel (version 2019). One-way ANOVA and Duncan’s multiple range test (*p* < 0.05) were used to evaluate the data differences. R software (version 2.9.1) was used to output the column diagram, Circos diagram, and hierarchical clustering analysis. The correlations between the dominant fungi and physicochemical properties were analyzed with the calculated Pearson’s correlation coefficients.

## 3. Results and Discussion

### 3.1. Physicochemical Properties of Suanyu

As shown in Table 2, the pH values of the Suanyu samples ranged between 4.30 and 5.50. The pH values of Suanyu samples made using inoculation fermentation ranged from 4.09 to 4.48 [18,22], which were lower than those of the Suanyu samples in this study. The average pH values of the G and H groups were 4.85 and 5.08, respectively, which were similar to that of the sample made using spontaneous fermentation in the study by Zeng et al. [22]. The low pH values mean that the accumulation of organic acids, which are essential in controlling the growth of spoilage and pathogens in fermented fish and fermented meats with a pH below 4.4, is usually considered safe, and fermented fish with mixed starter cultures could even be eaten uncooked [1]. However, the spontaneously fermented Suanyu was affected by the environmental microorganisms, and it was difficult to quickly reduce the pH values. Therefore, the selection of potential strains as starters for Suanyu is crucial.

The TVBN value is considered an index to evaluate the freshness of fish [23]. The content range of TVBN values was 17.11–94.70 mg/100 g, and H4 had the highest TVBN value of 94.70 mg/100 g. The average TVBN value in the G group was 26.81 mg/100 g, lower than that in the H group (67.94 mg/100 g), which exceeded the second-level freshness standard (≤25 mg/100 g), although the content of TVBN in the G group was still lower than the national standard (35 mg/100 g) and similar to the result found by Liu et al. [4]. In contrast, the content of TVBN in the H group exceeded the safety standard. This may have been due to the different fermentation environments.

TBARS are secondary products of lipid oxidation, and the TBARS value is used as an indicator of lipid oxidation in food products [24]. The acceptable maximum value of TBARS in fish products is 5 mg MDA/kg [1]. Except for H2, H6, and H7 samples, the TBARS contents (0.61 to 3.62 mg/kg) of other Suanyu samples were below the acceptable maximum value, and the average value was 1.96 mg/kg. In a previous study, the TBARS value of naturally fermented Suanyu was 2.11 mg/kg [4], while that of inoculation fermentation Suanyu was 2.27 mg/kg [9], which was higher than the majority samples in this study.

### 3.2. Biogenic Amine Contents in Suanyu

The contents of BAs in Suanyu are shown in Table 3. The predominant BAs found in the samples contained tryptamine (just H4), phenethylamine, putrescine, cadaverine, histamine, and tyramine, consistent with the result found by Han et al. [25]. Neither spermidine nor spermine was found in the samples. The G8 sample (4.51 mg/kg) had the lowest total BA content and the H7 sample (321.22 mg/kg) had the highest total BA content. Phenethylamine, putrescine, and cadaverine were the main BAs in Suanyu samples. High concentrations of BAs in fermented food can cause headache, hypertension, and other symptoms, and this issue has become one of the most pressing food safety concerns [15]. In previous studies, high levels of putrescine and cadaverine were found in other fermented fish products, such as Yucha [25], Yulu [26], and dry-salted fish [14]. In order to reduce the production of biogenic amines in Suanyu, strains without amino acid decarboxylase activity should be selected as starter cultures. Zeng et al. [16] found that the mixed starters suppressed the accumulation of putrescine in inoculation fermentation samples. The BA content has been suggested as a microbiological quality indicator, as foods containing excessive BAs may result in negative effects on humans [16]. BAs such as histamine and tyramine have long been considered toxic substances that impair the quality and safety of fishery foods [27]. Due to its toxicity, histamine is of special concern among BAs. The safety level of histamine is 50 mg/kg [25]. Tyramine concentrations higher than 125 mg/kg have undesirable effects in healthy individuals [28]. Histamine was mainly detected in the G group (G2, G3, G4, G6), and tyramine was mainly detected in the H group (H1, H2, H4, H5, H6, H7). As histamine and tyramine are mostly produced by microorganisms, the different contents of these two BAs in the G and H groups may be attributed to the different microbial communities.

### 3.3. Fungal Alpha Diversity Analysis of Suanyu Products

The fungal alpha diversity levels in different Suanyu products were analyzed and are shown in Table 4. After filtering, 1,025,940 ITS1 sequences were generated. These valid sequences were divided into different operational taxonomic units (OTUs) according to 97% similarity. The OTUs of the G group and H group were 864 and 746, respectively, resulting in a total of 1610 OTUs.

The diversity, richness, and Good’s coverage of the fungal community are shown in Table 4. The ACE and Chao1 indexes represent the richness of the microbial community and are used to estimate the total number of species in the sample. The community diversity is expressed by Shannon and Simpson indexes and shows the distribution characteristics of the species in the sample [29,30]. As shown in Table 4, the values of the alpha diversity indexes were diverse. Fungal richness and community diversity levels differed among samples. Good’s coverage, an indicator of sequencing completeness, was more than 0.99, meaning the fungi phylotypes in each sample were fully captured.

### 3.4. Fungal Community of Suanyu

**Figure 2:**
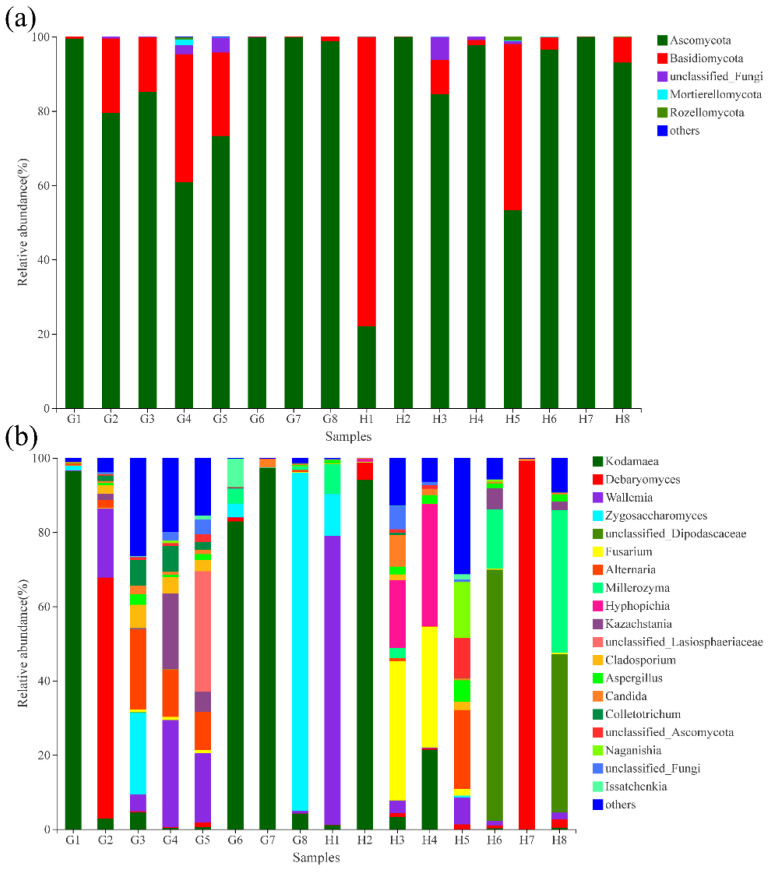
Relative abundance levels of fungi at the phylum (**a**) and genus (**b**) levels in the Suanyu samples. G1–G8: Suanyu samples from Guizhou; H1–H8: Suanyu samples from Hunan.

Figure 2 presents the relative abundance of the fungal composition at the phylum and genus levels. Ascomycota (53.25%–99.97%, H1 22.00%) was the dominant fungal phylum (Figure 2a). This result was consistent with that reported by Zang et al. [18]. Ascomycota also dominated in other traditional fermented foods, such as acid rice soup [31] and fermented dairy [32]. Basidiomycota was the second most dominant fungal phylum in all samples. It represented 77.87% of the total phyla in H1 and more than 5% in eight samples. Zang et al. [18] also detected Basidiomycota in the middle and late stages of Sunayu fermentation. Other phyla, such as Mortierello-mycota and Rozellomycota, were also observed. At the phylum level, the diversity of microorganism communities was simple and similar between different samples.

Figure 2b shows that 19 fungal genera were detected in Suanyu samples (relative abundance >5%). *Kodamaea*, *Debaryomyces*, *Wallemia*, *Zygosaccharomyces*, unclassified *Dipodascaceae*, and other genera were detected in different samples. The fungi in most samples belonged to yeasts. Many yeasts contribute to the flavor of fermented foods [33]. *Kodamaea* (82.84%–96.45%) was the dominant fungi in the G1, G6, G7, and H2 samples. Zhong et al. [34] found that *Kodamaea* produced acid, alcohol, and ethyl ester in sour meat. *Debaryomyces* was the dominant fungus in samples G2 and H7, accounting for 64.92% and 99.18%, respectively. *Debaryomyces* is commonly found in fermented fish [35] and meat sausages [36]. It may contribute to the lipolysis and proteolysis of fermented fish [35]. *Wallemia* (G2, G4, G5, H1, H5), *Zygosaccharomyces* (G3, G8, H1), unclassified *Dipodascaceae* (H6, H8), *Fusarium* (H3, H4), *Alternaria* (G3, G4, G5, H5), *Hyphopichia* (H3, H4), and unclassified *Lasiosphaeriaceae* (G5) were the dominant fungi in the different samples.

The results were compared with other Chinese traditional fermented foods to understand the uniqueness of fungal communities in Suanyu (Table 5). As shown in Table 5, the fermented products were divided into two categories: one based on meat, such as aquatic products (e.g., Suanyu) and fermented meat (e.g., sour meat), and the other based on grains and vegetables, such as condiments (e.g., soy sauce) and vegetables (e.g., Suancai). *Kodamaea* was the first dominant fungal genus in Suanyu, which was rarely found in other fermented food but is a characteristic fungi in Suanyu. The characteristic and predominant fungi are consistent with those in sour meat because of the similar fermentation process and place of origin. *Debaryomyces* was detected in almost all fermented foods, especially in condiments and vegetables; however, the abundance of *Debaryomyces* is higher in Suanyu than other fermented meats. *Pichia* and *Candida* are also common yeasts in fermented products, but the proportion in Suanyu was low. *Wallemia* and unclassified *Dipodascaceae* were only detected in Suanyu with high abundance.

### 3.5. Comparison of Fungal Communities of Suanyu from Two Regions

**Figure 3:**
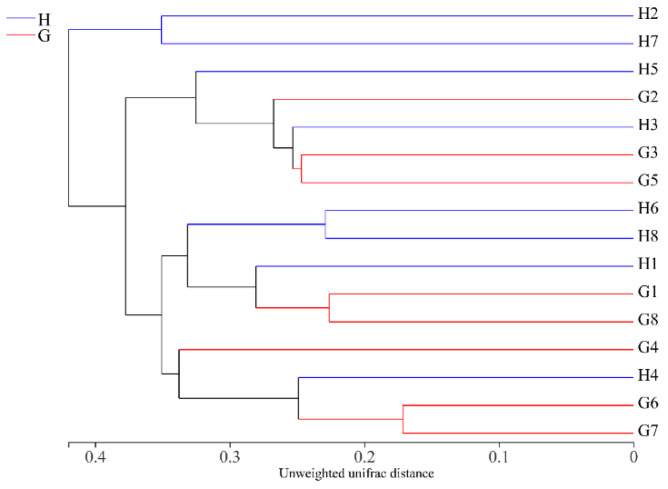
Unweighted unifrac clustering map of fungi in the Suanyu samples based on OTU pattern. Same clades: similar fungal composition. G1–G8: Suanyu samples from Guizhou; H1–H8: Suanyu samples from Hunan.

The fungal compositions of samples from different regions were compared via UPGMA clustering method based on the unweighted unifrac algorithm (Figure 3). The UPGMA tree was divided into four clades. Clade 1 included two samples (H2, H7), clade 2 had five samples (H3, H5, G2, G3, G5), clade 3 included five samples (H1, H6, H8, G1, G8), while the remaining four samples (G4, H4, G6, G7) were clustered into clade 4. The same clades indicates that they have similar fungal composition. Most samples from the same region were not clustered in a group.

**Figure 4:**
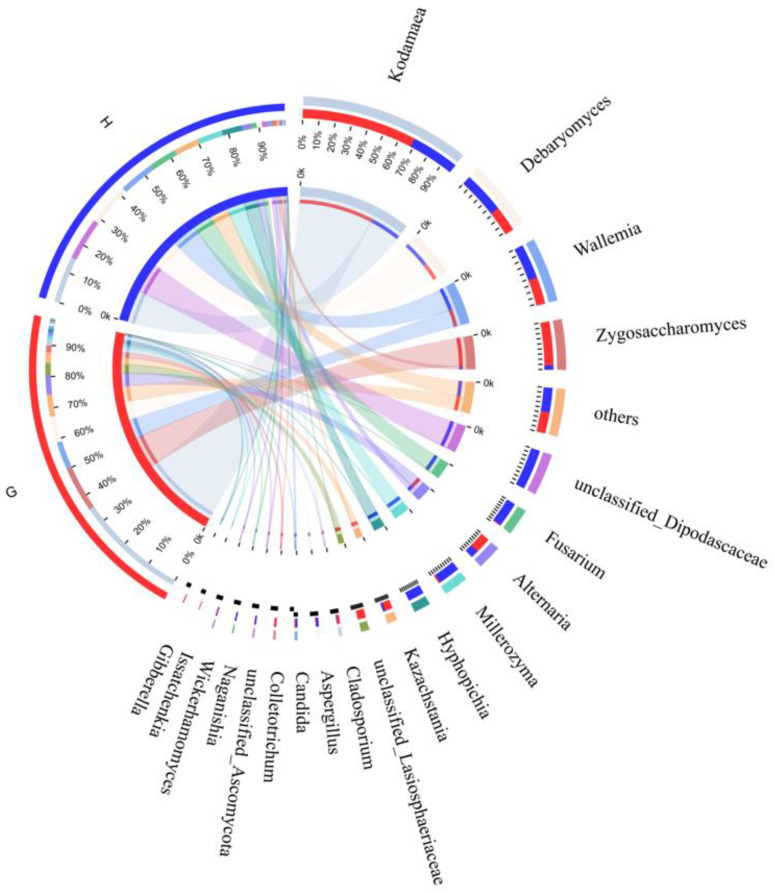
Circos diagram of fungi at the genus level in the Suanyu samples. G: The group of Suanyu samples from Guizhou; H: the group of Suanyu samples from Hunan.

To further clarify the differences in fungal communities between the two regions, the distribution proportions of dominant fungi in different Suanyu samples were studied through the Circos diagram (Figure 4). The relative abundance levels of *Kodamaea* (G: 71%, H: 29%) and *Zygosaccharomyces* (G: 91%, H: 9%) in the G group were higher than in the H group. *Debaryomyces* (G: 38%, H: 62%), *Wallemia* (G: 44%, H: 56%), and unclassified *Dipodascaceae* (G: 0.2%, H: 99.8%) made up a higher proportion in the G group than the H group.

### 3.6. Correlations between the Dominant Fungal Communities and Physicochemical Properties of Suanyu

**Figure 5:**
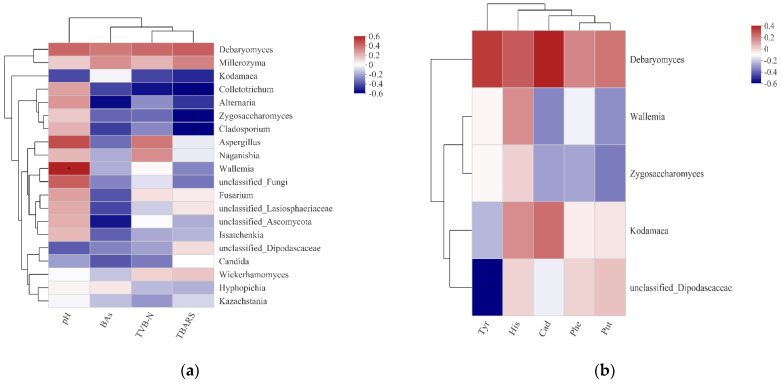
Correlation analysis between the fungal genera and physicochemical properties in the Suanyu samples (**a**). Correlation analysis between the fungal genera and five biogenic amines in the Suanyu samples (**b**). Note: * indicates *p* < 0.05; TVBN: total volatile base nitrogen; TBARS: thiobarbituric acid reactive substance; BAs: biogenic amines; Phe: phenethylamine; Put: putrescine, Cad: cadaverine; His: histamine; Tyr: tyramine.

The correlation between the abundance of fungal genera and physicochemical properties was visually displayed through the Spearman correlation heatmap, and the results are shown in Figure 5a. In the heat map, the colors red and blue represent positive and negative correlations, respectively. The heatmap showed that most of the dominant fungi were positively correlated with pH and negatively correlated with BAs, TVBN, and TBARS.

As shown in Figure 5a, *Kodamaea* was negatively correlated with pH (R^2^= −0.36), TBARS (R^2^ = −0.43), and TVBN (R^2^ = −0.37) and had a weak correlation with BAs. *Debaryomyces* was positively correlated with all physicochemical properties. *Zygosaccharomyces* was negatively correlated with TVBN (R^2^ = −0.29), TBARS (R^2^ = −0.51) and BAs (R^2^ = −0.38). *Wallemia* and unclassified *Dipodascaceae* were negatively correlated with BAs (R^2^ = −0.24, −0.15). In this study, *Kodamaea* and *Zygosaccharomyces* were the dominant fungi in traditional Suanyu, and the correlation results suggested that they may play an important role in the fermentation process. *Debaryomyces* is widely used in fermented foods, such as fermented meat and dairy products, and have also received attention as potential human probiotics [43]. However, *Debaryomyces* may cause an increase in the contents of TVBN, TBARS, and BAs during the fermentation of Suanyu according to this study, which may be caused by strain specificity.

The heatmap was used to further analyze the relationships between the five dominant fungal genera and five biogenic amines (Figure 5b). The results showed that *Zygosaccharomyces* was negatively correlated with phenethylamine (R^2^ = −0.26), putrescine (R^2^ = −0.34), and cadaverine (R^2^ = −0.27); unclassified *Dipodascaceae* was significant negatively correlated with tyramine (R^2^ = −0.57); *Wallemia* was negatively correlated with putrescine (R^2^ = −0.30) and cadaverine (R^2^ = −0.32); and *Debaryomyces* was positively correlated with all biogenic amines. Yeasts commonly found in fermented fish include *Debaryomyces*, *Candida,* and *Saccharomyces* [18,35,39]. Despite the yeasts contributing to flavor formation in the fermentation process of meat products [35], the correlation analysis results showed that *Debaryomyces* in fermented Suanyu was positively correlated with TVBN, TBARS, and BAs, which may impair the quality of Suanyu, while the abundance of *Candida* and *Saccharomyces* was low in Suanyu, which would have little effect on its quality. In a previous study, *Zygosaccharomyces* reduced the decarboxylase activity via an antagonism relationship with other yeasts, and the content of BAs was decreased [44]. In addition, Qi et al. [45] found that the expression of genes related to BA synthesis in *Zygosaccharomyces* may be related to the metabolic regulation of the microbial community, and the content of histamine was decreased because of the aldehyde dehydrogenase present in *Zygosaccharomyces*. *Kodamaea* and *Zygosaccharomyces* were found in traditional Suanyu samples and may play positive roles during the fermentation of Suanyu. This is the first time that these two yeasts have been detected in Suanyu.

## 4. Conclusions

In this study, the fungal community in Suanyu and its relationship with the physicochemical properties were studied using high-throughput sequencing technology. *Kodamaea*, *Debaryomyces*, *Wallemia*, *Zygosaccharomyces*, and unclassified *Dipodascaceae* were the dominant fungi in different samples, and most of the dominant fungi were yeast genera. Among the dominant fungi, *Kodamaea* and *Zygosaccharomyces* were negatively correlated with thiobarbituric acid reactive substance and total volatile base nitrogen values, and unclassified *Dipodascaceae* was significantly negatively correlated with tyramine. The contents of total volatile base nitrogen and biogenic amines in majority Suanyu samples exceeded the standards, and functional strains (such as the non-amino-acid decarboxylase active) could be screened for inoculation fermentation in the future to improve the fermentation quality. This study has provided insights about the fungal community of Suanyu and the fermentation characteristics of dominant fungi, which have important practical significance for the quality improvement of Suanyu.

## Figures and Tables

**Table 1:** Suanyu samples’ collection information.

Samples	Sampling Sites	Production Method	Sampling Time
G1	Tianzhu County, Guizhou	home-made	2020/10
G2	Liping County, Guizhou	home-made	2020/10
G3	Liping County, Guizhou	home-made	2020/10
G4	Liping County, Guizhou	home-made	2020/10
G5	Liping County, Guizhou	home-made	2020/10
G6	Rongjiang County, Guizhou	home-made	2020/10
G7	Congjiang County, Guizhou	home-made	2020/10
G8	Liping County, Guizhou	home-made	2020/10
H1	Guzhang County, Hunan	home-made	2020/10
H2	Guzhang County, Hunan	home-made	2020/10
H3	Jishou County, Hunan	home-made	2020/10
H4	Jishou County, Hunan	home-made	2020/10
H5	Guzhang County, Hunan	home-made	2020/10
H6	Jishou County, Hunan	home-made	2020/10
H7	Guzhang County, Hunan	home-made	2020/10
H8	Jishou County, Hunan	home-made	2020/10

G1–G8: Suanyu samples from Guizhou (103°36′ E–109°35′ E, 24°37′ N–29°13′ N); H1–H8: Suanyu samples from Hunan (108°47′ E–114°15′ E, 24°38′ N–30°08′ N).

**Table 2:** The pH values and TVB-N and TBARS contents of Suanyu samples.

Samples	pH	TVBN (mg/100 g)	TBARS (mg/kg)
G1	4.52 ± 0.01 ^l^	32.86 ± 0.81 ^f^	0.72 ± 0.01 ^m^
G2	5.34 ± 0.01 ^c^	33.43 ± 0.00 ^f^	0.61 ± 0.02 ^m^
G3	4.81 ± 0.01 ^i^	27.82 ± 0.00 ^h^	1.53 ± 0.02 ^kl^
G4	4.55 ± 0.01 ^k^	17.11 ± 0.81 ^j^	1.65 ± 0.11 ^jk^
G5	5.29 ± 0.01 ^d^	32.05 ± 0.00 ^f^	2.32 ± 0.07 ^g^
G6	4.96 ± 0.00 ^f^	28.17 ± 6.43 ^gh^	1.41 ± 0.04 ^l^
G7	4.30 ± 0.00 ^m^	20.47 ± 1.37 ^ij^	2.12 ± 0.10 ^h^
G8	5.07 ± 0.00 ^e^	22.58 ± 0.80 ^i^	1.79 ± 0.13 ^i^
H1	5.48 ± 0.00 ^b^	78.96 ± 1.59 ^c^	2.05 ± 0.03 ^h^
H2	4.97 ± 0.00 ^f^	34.65 ± 1.39 ^f^	19.72 ± 0.17 ^a^
H3	5.29 ± 0.01 ^d^	31.47 ± 3.64 ^fg^	2.90 ± 0.02 ^f^
H4	4.87 ± 0.00 ^h^	94.70 ± 1.39 ^a^	1.73 ± 0.04 ^ij^
H5	5.50 ± 0.00 ^a^	86.17 ± 0.69 ^b^	3.62 ± 0.08 ^d^
H6	4.94 ± 0.00 ^g^	68.23 ± 0.40 ^e^	5.81 ± 0.02 ^c^
H7	4.63 ± 0.00 ^j^	72.46 ± 0.40 ^d^	10.18 ± 0.02 ^b^
H8	4.97 ± 0.02 ^f^	76.85 ± 0.40 ^c^	3.08 ± 0.04 ^e^

Data are expressed as means ± standard deviations (*n* = 3). The same superscript letters in a column indicate no significant differences (*p* > 0.05). TVBN: total volatile base nitrogen; TBARS: thiobarbituric acid reactive substance. G1–G8: Suanyu samples from Guizhou; H1–H8: Suanyu samples from Hunan.

**Table 3:** Contents of BAs in Suanyu samples (mg/kg fresh weight).

Samples	Tryptamine	Phenethylamine	Putrescine	Cadaverine	Histamine	Tyramine	Spermidine	Spermine	Total Content
G1	ND	23.81 ± 1.30 ^c^	36.29 ± 1.94 ^g^	18.76 ± 1.28 ^g^	ND	7.14 ± 0.72 ^e^	ND	ND	86.00 ± 5.20 ^f^
G2	ND	24.84 ± 1.22 ^bc^	13.64 ± 0.90 ^j^	96.19 ± 5.64 ^b^	57.12 ± 3.79 ^a^	14.70 ± 1.47 ^c^	ND	ND	206.49 ± 13.00 ^c^
G3	ND	7.21 ± 0.70 ^g^	12.13 ± 0.63 ^j^	8.12 ± 1.19 ^hi^	1.06 ± 0.05 ^d^	ND	ND	ND	29.97 ± 0.21 ^i^
G4	ND	6.54 ± 0.36 ^gh^	ND	ND	39.97 ± 0.57 ^b^	ND	ND	ND	46.51 ± 0.81 ^h^
G5	ND	4.65 ± 0.11 ^i^	ND	ND	ND	ND	ND	ND	4.65 ± 0.11 ^k^
G6	ND	15.28 ± 0.22 ^d^	22.23 ± 0.43 ^i^	32.60 ± 0.56 ^f^	15.93 ± 0.46 ^c^	ND	ND	ND	86.04 ± 1.65 ^f^
G7	ND	ND	ND	5.70 ± 0.45 ^ij^	ND	ND	ND	ND	5.70 ± 0.45 ^k^
G8	ND	ND	0.66 ± 0.56 ^k^	3.85 ± 0.64 ^j^	ND	ND	ND	ND	4.51 ± 0.19 ^k^
H1	ND	5.60 ± 0.43 ^hi^	95.40 ± 0.89 ^b^	39.82 ± 0.68 ^e^	ND	75.04 ± 0.50 ^b^	ND	ND	215.85 ± 2.45 ^b^
H2	ND	ND	57.64 ± 0.94 ^d^	100.96 ± 1.31 ^a^	2.45 ± 0.28 ^d^	12.28 ± 0.37 ^d^	ND	ND	173.32 ± 2.86 ^d^
H3	ND	13.75 ± 0.45 ^e^	24.27 ± 0.93 ^h^	44.74 ± 1.47 ^d^	ND	ND	ND	ND	82.76 ± 2.83 ^f^
H4	4.83 ± 0.02 ^a^	25.22 ± 0.49 ^b^	88.91 ± 1.77 ^c^	32.18 ± 0.75 ^f^	ND	13.95 ± 0.47 ^c^	ND	ND	165.09 ± 3.47 ^e^
H5	ND	ND	ND	ND	ND	14.45 ± 1.47 ^c^	ND	ND	14.45 ± 1.47 ^j^
H6	ND	9.44 ± 0.44 ^f^	57.92 ± 1.32 ^e^	16.58 ± 0.81 ^g^	ND	2.69 ± 0.28 ^f^	ND	ND	81.64 ± 2.71 ^f^
H7	ND	35.18 ± 0.20 ^a^	131.59 ± 0.22 ^a^	65.15 ± 0.08 ^c^	ND	89.30 ± 0.47 ^a^	ND	ND	321.22 ± 0.81 ^a^
H8	ND	5.40 ± 0.65 ^hi^	43.73 ± 1.17 ^f^	11.06 ± 0.39 ^h^	ND	ND	ND	ND	60.19 ± 2.17 ^g^

All values are expressed as the means (*n* = 3) ± standard deviations. The same superscript letters in a column indicate no significant differences (*p* > 0.05). ND, not detected. G1–G8: Suanyu samples from Guizhou; H1–H8: Suanyu samples from Hunan. Detection limit for each amine (mg/kg): tryptamine: 0.18; phenethylamine: 0.25; putrescine: 0.30; cadaverine: 0.28; histamine: 0.45; tyramine: 0.25; spermidine: 0.30; spermine: 0.65.

**Table 4:** Fungal alpha diversity levels in Suanyu samples from different regions.

Samples ID	Sequence Number	OTUs Observed	Ace	Chao1	Shannon	Simpson	Good’s Coverage
G1	73295	61	66.528	65.000	0.700	0.699	0.999
G2	50273	99	99.946	99.500	1.495	0.454	0.999
G3	51316	152	152.907	152.375	3.291	0.078	0.999
G4	63390	228	235.139	243.111	2.900	0.127	0.999
G5	68600	131	135.551	138.000	2.738	0.153	0.999
G6	70596	59	88.824	74.300	0.903	0.640	0.999
G7	72324	42	52.660	50.250	0.616	0.686	0.999
G8	52813	92	108.407	103.667	1.395	0.353	0.999
H1	72509	83	114.963	114.500	1.298	0.476	0.998
H2	71542	38	77.762	55.500	1.144	0.382	0.999
H3	65711	145	146.072	146.500	2.631	0.180	0.999
H4	73885	133	147.692	142.130	1.917	0.242	0.999
H5	51915	89	89.353	89.000	3.332	0.061	0.999
H6	71840	130	142.133	139.500	3.332	0.061	0.999
H7	44641	15	165.730	33.000	0.056	0.984	0.999
H8	71290	113	123.370	122.714	1.606	0.332	0.999

G1–G8: Suanyu samples from Guizhou; H1–H8: Suanyu samples from Hunan.

**Table 5:** Comparison of Suanyu with other Chinese traditional fermented foods.

Chinese Fermented Food		Materials	Dominant Fungi (Genus Level)	References
Aquatic products	Suanyu	carp, rice or roasted millet, salt	*Kodamaea*, *Debaryomyces*, *Wallemia*, *Zygosaccharomyces*, unclassified *Dipodascaceae*	this research
	Suanzhayu	carp, rice flour, salt	*Saccharomyces*, *Candida*, *Apiotrichum*, *Trichosporon*, *Debaryomyces*	[37]
	Shrimp paste	shrimp, salt	*Aspergillus*, unclassified*_k_Fungi*, *Monascus*	[38]
Meat	Sour meat	pork, fried rice, salt	*Kodamaea*, *Hyphopichia*, unclassified *Saccharomycetales*	[34]
	Sausage	pork belly, millet, salt	*Pichia*, *Candida*, *Kazachstania*, *Issatchenkia*, *Debaryomyces*	[36,39]
Condiment	Soy sauce/Shoyu	soybeans, wheat	*Candid, Wickerhamiella Zygosaccharomyces Millerozyma Debaryomyces*	[33,40]
	Sour soup	rice, wild cherry tomato	*Candida*, *Pichia*, *Saccharomyces, Debaryomyces,Kazachstania*,	[31,41]
Vegetables	Suansun/Suancai	bamboo shoots, radish, cabbage	*Kazachstania*, *Debaryomyces*, *Pichia*, *Nakaseomyces*	[42]

## Data Availability

The data presented in this study are available on request from the corresponding author.
